# Mapping algorithms for predicting EuroQol-5D-3L utilities from the assessment test of chronic obstructive pulmonary disease

**DOI:** 10.1038/s41598-022-24956-2

**Published:** 2022-12-03

**Authors:** Chun-Hsiang Yu, Sheng-Mao Chang, Chih-Hui Hsu, Sheng-Han Tsai, Xin-Min Liao, Chang-Wei Chen, Ching-Hsiung Lin, Jung-Der Wang, Tzuen-Ren Hsiue, Chiung-Zuei Chen

**Affiliations:** 1 Division of Pulmonary Medicine, Department of Internal Medicine, College of Medicine, National Cheng Kung University Hospital, National Cheng Kung University, No. 138 Sheng-Li Road, 704 Tainan, Taiwan; 2 Department of Statistics, National Taipei University, New Taipei, Taiwan; 3 Clinical Medicine Research Center, National Cheng Kung University Hospital, Tainan, Taiwan; 4 Division of General Medicine, Department of Internal Medicine, College of Medicine, National Cheng Kung University Hospital, National Cheng Kung University, Tainan, Taiwan; 5 Division of Chest Medicine, Department of Internal Medicine, Changhua Christian Hospital, Changhua, Taiwan; 6 Department of Public Health, College of Medicine, National Cheng Kung University, Tainan, Taiwan

**Keywords:** Diseases, Health care, Medical research

## Abstract

To predict 3-Level version of European Quality of Life-5 Dimensions (EQ-5D-3L) questionnaire utility from the chronic obstructive pulmonary disease (COPD) assessment test (CAT), the study attempts to collect EQ-5D-3L and CAT data from COPD patients. Response mapping under a backward elimination procedure was used for EQ-5D score predictions from CAT. A multinomial logistic regression (MLR) model was used to identify the association between the score and the covariates. Afterwards, the predicted scores were transformed into the utility. The developed formula was compared with ordinary least squares (OLS) regression models and models using Mean Rank Method (MRM). The MLR models performed as well as other models according to mean absolute error (MAE) and root mean squared error (RMSE) evaluations. Besides, the overestimation for low utility patients (utility ≤ 0.6) and underestimation for near health (utility > 0.9) in the OLS method was improved through the means of the MLR model based on bubble chart analysis. In conclusion, response mapping with the MLR model led to performance comparable to the OLS and MRM models for predicting EQ-5D utility from CAT data. Additionally, the bubble charts analysis revealed that the model constructed in this study and MRM could be a better predictive model.

## Introduction

Quality of life-related to chronic obstructive pulmonary disease (COPD) is impaired, and it deteriorates significantly with increases in severity in COPD patients^1^. In addition to causing disabilities, the disease generates high healthcare costs and heavy socioeconomic burdens^2^. In 2013, the COPD prevalence in people aged over 40 years was 6.1% in Taiwan^3^, and it was the seventh leading cause of death in 2018. The estimated loss of life expectancy in patients at moderate and severe stages of the disease was 6.2 and 9.4 years, respectively^4^.

COPD-specific health-related quality of life instruments, including the CAT (COPD Assessment Test), CCQ (Clinical COPD Questionnaire), and SGRQ (St George's Respiratory Questionnaire), were designed to reliably assess the impact of the disease on patients. The CCQ and CAT have the advantage of being simpler to administer^5^. Furthermore, the CAT has been introduced as a tool for differentiating the severity of COPD in patients^6^. The usage of this questionnaire has been also extended to many clinical studies and practice guidelines^7^. The short and self-administered eight-item questionnaire consists of coughing, phlegm, chest tightness, breathlessness, home activities, leaving home, sleep problems, and energy. Each item has six levels ranked on a scale ranging from 0 to 5. Therefore, the total CAT scores will range from 0 to 40.

The CAT cannot be used directly for quality adjustments in the measurement of quality-adjusted life-years in a cost-utility analysis^8^. Therefore, studies using mapping algorithms to estimate European Quality of Life-5 Dimensions (EQ-5D) utilities from the CAT were investigated^9,10^. However, overestimated utilities were reported in COPD patients at advanced stages of the disease using the previous mapping algorithms from CAT scores, developed by Hoyle et al. in the UK^9^. A similar situation occurred by using the mapping algorithms, created by Lim et al. in South Korea and even greater underestimations of utilities were found for mild COPD cases^10^.. The aim of this study was thus to explore a suitable mapping algorithm for COPD patients in Taiwan.

## Methods

The Institutional Review Board of National Cheng Kung University Hospital (NCKUH) approved this study before commencement (IRB number: B-ER-98-289 and B-ER-111-254). Informed consent was obtained from all subjects, and all methods were carried out in accordance with the relevant guidelines and regulations of the research ethics committee.

In this study, 323 patients were enrolled who were diagnosed with COPD in the outpatient Pulmonary Medicine Clinic at National Cheng Kung University Medical Center from April 2017 to December 2020. All patients were enrolled in the pay-for-performance program of COPD and had been receiving regular medical treatment for COPD for more than three months. These COPD cases were defined according to the GOLD diagnosis guideline and criteria^7^. All pulmonary function tests were performed according to a joint consensus of the American Thoracic Society and the European Respiratory Society^11^. Patients who were unwilling to participate, unable to receive the pay-for-performance program (for example, bed-ridden), or had advanced lung cancer and pulmonary fibrosis were excluded.

Participants were classified using the GOLD 2017 classification and were divided into four stages (mild to very severe), which corresponded to the GOLD 2017 grades 1 to 4, based on the post-bronchodilator forced expiratory volume in one second (FEV_1_): grade 1 or mild stage (FEV_1_ ≥ 80%), grade 2 or moderate stage (50% ≤ FEV_1_ < 80%), grade 3 or severe stage (30% ≤ FEV_1_ < 50%) and grade 4 or very severe stage (FEV_1_ < 30%)^7^. In this study, the participants with FEV_1_ < 50% were incorporated into the “severe” category to obtain a sufficient number in the sample for estimation.

The quality of life of the COPD patients was consistently monitored with the EQ-5D-3L and the CAT in order to develop an algorithm for estimating EQ-5D equivalent utilities from the CAT. The Validated Taiwanese version of EQ-5D-3L and Chinese version of CAT questionnaires were used in this study^12,13^.

The questionnaires were administered by the case manager of the pay-for-performance program at the outpatient department.

### Model development

The COPD patient dataset was randomly split into a training group of 160 patients and a validation group of 163 patients. While the predictive model was built, the coefficients of the final model were derived based on the full sample (all 323 patients) to get the most accurate estimates. In this study, we considered two OLS-based procedures to build predictive models for COPD patients. The first was the model recommended by Hoyle et al.^9^. They regressed the EQ-5D utility on 8 CAT scores and chose 4 CAT scores (chest tightness, activities, confidence, and energy) with *p*-values smaller than 0.05 to build the final model. We created modified versions of the models by Hoyle et al. and Lim et al. that better fit the Taiwanese population. A backward elimination procedure was applied to obtain a final parsimonious model with a type I error rate of 0.05 when statistical hypothesis testing was performed.

Response mapping is another feasible approach that can be used for utility prediction^14,15^. While OLS is aimed toward predict EQ-5D utility, response mapping is aimed toward predict five EQ-5D scores, each taking values of 1, 2, or 3. The five predicted scores are then transformed to the utility. The transformation formula varies across different underlying populations. In this study, the formula, which is based on Taiwanese population obtained from Lee et al. was applied^16^. The formula was: EQ-5D-3L utility = 1–0.185–0.123*Mobility at level 2–0.272*Mobility at level 3–0.167*Self-care at level 2–0.276*Self-care at level 3–0.085*Usual activities at level 2–0.208*Usual activities at level 3–0.121*Pain/discomfort at level 2–0.261*Pain/discomfort at level 3–0.154*Anxiety/depression at level 2–0.282*Anxiety/depression at level 3–0.190*Any dimension on level 3.

Furthermore, because the EQ-5D score takes discrete values, a multinomial logistic regression (MLR) is a relevant model to use to identify the association between the score and covariates, 8 CAT scores, age, and sex. In our dataset, most of patients filled out both the EQ-5D and CAT questionnaires multiple times during the follow-up period. In other words, the experiment consisted of repeated measurements. Therefore, a generalized estimating equation (GEE) was applied with an independent working correlation for estimation and hypothesis testing^17^. For each EQ-5D score prediction, the final multinomial logistic regression was chosen so that the resulting QIC was minimized^18^. The GEE models were performed using the SAS GEE procedure.

We also applied the Mean Rank Method (MRM), developed by Wee, et al.^19^, as the other method for developing a predictive model of mapping EQ-5D utilities from CAT. The MRM considers nonparametric matching among EQ-5D and CAT scores, preventing potentially erroneous model assumptions and providing less interpretation information.

### Validation

Among all applied methods, OLS, MLR, and MRM, the training data was used to build predictive models, whereas the validation data was used to evaluate the performance of these models via both the root mean squared error (RMSE) and mean absolute error (MAE). In addition, the models of Hoyle et al. and Lim et al. were modified by re-estimating their coefficients using the training group and validated by the validation group for comparison.

In this study, as expected, the model with the best predicting ability should have the smallest RMSE and MAE. Additionally, to visualize the potential prediction biases, we suggest the bubble chart drawn with R version 4.2.1statistical software, R Core Team (2021). R: A language and environment for statistical computing. R Foundation for Statistical Computing, Vienna, Austria. URL https://www.R-project.org/ in this paper. The best model should locate a majority of bubbles on the diagonal line of the bubble chart. As for statistical comparisons among groups, continuous variables were analyzed by t-test, and the categorical variables were analyzed by chi-square. All statistical analyses were conducted by using SAS version 9.4 statistical software (SAS Institute Inc., Cary, NC, USA).

### Ethics approval

The Institutional Review Board of National Cheng Kung University Hospital (NCKUH) approved this study before commencement (IRB number: B-ER-98-289 and B-ER-111-254).

## Results

In this study, 323 patients were recruited, and 2327 repeated measures were done. The mean number of visits for each patient was seven. The range of visit frequency was from one to sixteen times. Overall, the mean EQ-5D-3L utility and CAT total scores data were 0.917 and 9.88, respectively. Between the training and the validation group, the EQ-5D-3L utility and CAT total scores were similar at baseline. No differences were revealed over the distribution of patient characteristics (supplementary Table 1).

A negative correlation (-0.69) between EQ-5D-3L utility and the total CAT score was observed (Fig. S1). The distribution of the EQ-5D-3L utility and CAT scores from all eligible patients are presented in Fig. S2. The largest cluster was located at EQ-5D utility = 1: n = 1624 (69.79% of observations). The other clusters were 0.5 ≤ EQ-5D < 1: n = 638 (27.42%) and 0 ≤ EQ-5D < 0.5: n = 65 (2.79%).

### Model development

Two MLR models were produced from training group using either the total CAT score or selected CAT items, the coefficients of the final model were derived based on all 323 patients to get the most accurate estimates (supplementary Table 2). The formula for developed models in this study, including the models developed by Hoyle et al. and Lim et al. and their modified versions are presented in Table 1. Estimated utility scores were presented with mean, maximum, and minimum scores for each model with subsets of the EQ-5D utility values, CAT scores, or FEV_1_ stages. The RMSE and MAE were calculated for each subset to examine the distribution of errors across the different disease severities (Table 2). An overestimation of the mean EQ-5D was presented among the patients categorized as having poor health (0 ≤ EQ-5D < 0.25 and 0.25 ≤ EQ-5D < 0.5) in both models.

**Table 1:** Formula for current developed models.

Models	Formula for the developed model
Present study using CAT total score model	Probability (predicted Mobility = 2) = $$\frac{\mathrm{exp}(-13.79 + 0.37\mathrm{ x CAT total scores }+ 0.10\mathrm{ x age }+ 0.64\mathrm{ x sex})}{\begin{array}{c}1 + \mathrm{exp}\left(-13.79 + 0.37\mathrm{ x CAT total scores }+ 0.10\mathrm{ x age }+ 0.64\mathrm{ x sex}\right) +\\ exp(-43.98 + 0.52 x CAT total scores + 0.27 x age + 10.33 x sex)\end{array}}$$ Probability (predicted Mobility = 3) = $$\frac{\mathrm{exp}(-43.98 + 0.52\mathrm{ x CAT total scores }+ 0.27\mathrm{ x age }+ 10.33\mathrm{ x sex})}{\begin{array}{c}1 + \mathrm{exp}\left(-13.79 + 0.37\mathrm{ x CAT total scores }+ 0.10\mathrm{ x age }+ 0.64\mathrm{ x sex}\right)+\\ exp(-43.98 + 0.52 x CAT total scores + 0.27 x age + 10.33 x sex)\end{array}}$$ Probability (predicted Mobility = 1) = 1–Probability (predicted Mobility = 2)–Probability (predicted Mobility = 3) predicted Mobility, choosing if maximum predicted probability of Mobility Probability (predicted Self-care = 2) = $$\frac{\mathrm{exp}(-14.04 + 0.40\mathrm{ x CAT total scores }+ 0.06\mathrm{ x age }+ 3.11\mathrm{ x sex})}{\begin{array}{c}1 + \mathrm{exp}\left(-14.04 + 0.40\mathrm{ x CAT total scores }+ 0.06\mathrm{ x age }+ 3.11\mathrm{ x sex}\right) +\\ exp(-35.06 + 0.59 x CAT total scores + 0.13 x age + 13.91 x sex)\end{array}}$$ Probability (predicted Self-care = 3) = $$\frac{\mathrm{exp}(-35.06 + 0.59\mathrm{ x CAT total scores }+ 0.13\mathrm{ x age }+ 13.91\mathrm{ x sex})}{\begin{array}{c}1 + \mathrm{exp}\left(-14.04 + 0.40\mathrm{ x CAT total scores }+ 0.06\mathrm{ x age }+ 3.11\mathrm{ x sex}\right) +\\ exp(-35.06 + 0.59 x CAT total scores + 0.13 x age + 13.91 x sex)\end{array}}$$ Probability (predicted Self-care = 1) = 1–Probability (predicted Self-care = 2)–Probability (predicted Self-care = 3) predicted Self-care, choosing if maximum predicted probability of Self-care Probability (predicted Usual activities = 2) = $$\frac{\mathrm{exp}(-15.37 + 0.48\mathrm{ x CAT total scores }+ 0.09\mathrm{ x age }+ 2.04\mathrm{ x sex})}{\begin{array}{c}1 + \mathrm{exp}\left(-15.37 + 0.48\mathrm{ x CAT total scores }+ 0.09\mathrm{ x age }+ 2.04\mathrm{ x sex}\right) +\\ exp(-37.84 + 0.75 x CAT total scores + 0.14 x age + 13.42 x sex)\end{array}}$$ Probability (predicted Usual activities = 3) = $$\frac{\mathrm{exp}(-37.84 + 0.75\mathrm{ x CAT total scores }+ 0.14\mathrm{ x age }+ 13.42\mathrm{ x sex})}{\begin{array}{c} 1 + \mathrm{exp}\left(-15.37 + 0.48\mathrm{ x CAT total scores }+ 0.09\mathrm{ x age }+ 2.04\mathrm{ x sex}\right) +\\ exp(-37.84 + 0.75 x CAT total scores + 0.14 x age + 13.42 x sex)\end{array}}$$ Probability (predicted Usual activities = 1) = 1–Probability (predicted Usual activities = 2)–Probability (predicted Usual activities = 3) predicted Usual activities, choosing if maximum predicted probability of Usual activities Probability (predicted Pain / discomfort = 2) = $$\frac{\mathrm{exp}(-5.61 + 0.30\mathrm{ x CAT total scores }+ 0.01\mathrm{ x age }- 0.84\mathrm{ x sex})}{\begin{array}{c}1 + \mathrm{exp}\left(-5.61 + 0.30\mathrm{ x CAT total scores }+ 0.01\mathrm{ x age }-0.84\mathrm{ x sex}\right) +\\ exp(-31.06 + 0.79 x CAT total scores + 0.02 x age + 8.77 x sex)\end{array}}$$ Probability (predicted Pain / discomfort = 3) = $$\frac{\mathrm{exp}(-31.06 + 0.79\mathrm{ x CAT total scores }+ 0.02\mathrm{ x age }+ 8.77\mathrm{ x sex})}{\begin{array}{c}1 + \mathrm{exp}\left(-5.61 + 0.30\mathrm{ x CAT total scores }+ 0.01\mathrm{ x age }- 0.84\mathrm{ x sex}\right) +\\ exp(-31.06 + 0.79 x CAT total scores + 0.02 x age + 8.77 x sex)\end{array}}$$ Probability (predicted Pain / discomfort = 1) = 1–Probability (predicted Pain / discomfort = 2)–Probability (predicted Pain / discomfort = 3) predicted Pain / discomfort, choosing if maximum predicted probability of Pain / discomfort Probability (predicted Anxiety / depression = 2) = $$\frac{\mathrm{exp}(-4.07 + 0.28\mathrm{ x CAT total scores }- 0.02\mathrm{ x age }- 0.41\mathrm{ x sex})}{\begin{array}{c}1 + \mathrm{exp}\left(-4.07 + 0.28\mathrm{ x CAT total scores }- 0.02\mathrm{ x age }- 0.41\mathrm{ x sex}\right) +\\ exp(-25.77 + 1.39 x CAT total scores - 0.22 x age + 3.75 x sex)\end{array}}$$ Probability (predicted Anxiety / depression = 3) = $$\frac{\mathrm{exp}(-25.77 + 1.39\mathrm{ x CAT total scores }- 0.22\mathrm{ x age }+ 3.75\mathrm{ x sex})}{\begin{array}{c}1 + \mathrm{exp}\left(-4.07 + 0.28\mathrm{ x CAT total scores }- 0.02\mathrm{ x age }- 0.41\mathrm{ x sex}\right) +\\ exp(-25.77 + 1.39 x CAT total scores - 0.22 x age + 3.75 x sex)\end{array}}$$ Probability (predicted Anxiety / depression = 1) = 1–Probability (predicted Anxiety / depression = 2)–Probability (predicted Anxiety / depression = 3) predicted Anxiety / depression, choosing if maximum predicted probability of Anxiety / depression **Predicted utility** = 1–0.185–0.123 × predicted Mobility at level 2–0.272 × predicted Mobility at level 3–0.167 × predicted Self-care at level 2–0.276 × predicted Self-care at level 3–0.085 × predicted Usual activities at level 2–0.208 × predicted Usual activities at level 3–0.121 × predicted Pain / discomfort at level 2–0.261 × predicted Pain / discomfort at level 3–0.154 × predicted Anxiety / depression at level 2–0.282 × predicted Anxiety / depression at level 3–0.190 × Any dimension on level 3
Present study using CAT selected items model	Probability (predicted Mobility = 2) = $$\frac{\mathrm{exp}(-10.89 - 0.46\mathrm{ x Q}2 - 0.04\mathrm{ x Q}3 + 0.60\mathrm{ x Q}4 + 0.63\mathrm{ x Q}5 + 1.19\mathrm{ x Q}6 + 0.39\mathrm{ x Q}8 + 0.07\mathrm{ x age})}{\begin{array}{c}1 +\mathrm{ exp}\left(-10.89 - 0.46\mathrm{ x Q}2 - 0.04\mathrm{ x Q}3 + 0.60\mathrm{ x Q}4 + 0.63\mathrm{ x Q}5 + 1.19\mathrm{ x Q}6 + 0.39\mathrm{ x Q}8 + 0.07\mathrm{ x age}\right)+\\ exp(-37.51 - 0.92 x Q2 + 1.26 x Q3 + 0.74 x Q4 + 0.47 x Q5 + 1.74 x Q6 + 0.01 x Q8 + 0.31 x age)\end{array}}$$ Probability (predicted Mobility = 3) = $$\frac{\mathrm{exp}(-37.51 - 0.92\mathrm{ x Q}2 + 1.26\mathrm{ x Q}3 + 0.74\mathrm{ x Q}4 + 0.47\mathrm{ x Q}5 + 1.74\mathrm{ x Q}6 + 0.01\mathrm{ x Q}8 + 0.31\mathrm{ x age})}{\begin{array}{c}1 + \mathrm{exp}\left(-10.89 - 0.46\mathrm{ x Q}2 - 0.04\mathrm{ x Q}3 + 0.60\mathrm{ x Q}4 + 0.63\mathrm{ x Q}5 + 1.19\mathrm{ x Q}6 + 0.39\mathrm{ x Q}8 + 0.07\mathrm{ x age}\right) +\\ exp(-37.51 - 0.92 x Q2 + 1.26 x Q3 + 0.74 x Q4 + 0.47 x Q5 + 1.74 x Q6 + 0.01 x Q8 + 0.31 x age)\end{array}}$$ Probability (predicted Mobility = 1) = 1–Probability (predicted Mobility = 2)–Probability (predicted Mobility = 3) predicted Mobility, choosing if maximum predicted probability of Mobility Probability (predicted Self-care = 2) = $$\frac{\begin{array}{c}exp(-9.21 -0.39 x Q2 + 1.30 x Q5 + 1.11 x Q6 + 0.35 x Q7 + 0.03 x age + 1.98 x sex)\end{array}}{\begin{array}{c}1 + \mathrm{exp}\left(\begin{array}{c}-9.21 - 0.39 x Q2 + 1.30 x Q5 + 1.11 x Q6 + 0.35 x Q7 + 0.03 x age + 1.98 x sex\end{array}\right) +\\ exp(-29.54 - 0.53 x Q2 + 1.80 x Q5 + 1.47 x Q6 + 0.79 x Q7 + 0.09 x age + 12.68 x sex)\end{array}}$$ Probability (predicted Self-care = 3) = $$\frac{\begin{array}{c}exp(-29.54 - 0.53 x Q2 + 1.80 x Q5 + 1.47 x Q6 + 0.79 x Q7 + 0.09 x age + 12.68 x sex)\end{array}}{\begin{array}{c}1 + \mathrm{exp}\left(-9.21 - 0.39\mathrm{ x Q}2 + 1.30\mathrm{ x Q}5 + 1.11\mathrm{ x Q}6 + 0.35\mathrm{ x Q}7 + 0.03\mathrm{ x age }+ 1.98\mathrm{ x sex}\right) +\\ exp(-29.54 - 0.53 x Q2 + 1.80 x Q5 + 1.47 x Q6 + 0.79 x Q7 + 0.09 x age + 12.68 x sex)\end{array}}$$ Probability (predicted Self-care = 1) = 1–Probability (predicted Self-care = 2)–Probability (predicted Self-care = 3) predicted Self-care, choosing if maximum predicted probability of Self-care Probability (predicted Usual activities = 2) = $$\frac{\begin{array}{c}exp(-11.90 + 0.28 x Q3 + 0.43 x Q4 + 1.08 x Q5 + 1.52 x Q6 + 0.25 x Q7 + 0.06 x age + 0.98 x sex)\end{array}}{\begin{array}{c}1 + \mathrm{exp}\left(-11.90 + 0.28\mathrm{ x Q}3 + 0.43\mathrm{ x Q}4 + 1.08\mathrm{ x Q}5 + 1.52\mathrm{ x Q}6 + 0.25\mathrm{ x Q}7 + 0.06\mathrm{ x age }+ 0.98\mathrm{ x sex}\right) +\\ exp(-33.79 + 0.53 x Q3 + 0.37 x Q4 + 2.37 x Q5 + 1.68 x Q6 + 0.57 x Q7 + 0.11 x age + 11.41 x sex)\end{array}}$$ Probability (predicted Usual activities = 3) = $$\frac{\begin{array}{c}exp(-33.79 + 0.53 x Q3 + 0.37 x Q4 + 2.37 x Q5 + 1.68 x Q6 + 0.57 x Q7 + 0.11 x age + 11.41 x sex)\end{array}}{\begin{array}{c}1 + \mathrm{exp}\left(-11.90 + 0.28\mathrm{ x Q}3 + 0.43\mathrm{ x Q}4 + 1.08\mathrm{ x Q}5 + 1.52\mathrm{ x Q}6 + 0.25\mathrm{ x Q}7 + 0.06\mathrm{ x age }+ 0.98\mathrm{ x sex}\right) +\\ exp(-33.79 + 0.53 x Q3 + 0.37 x Q4 + 2.37 x Q5 + 1.68 x Q6 + 0.57 x Q7 + 0.11 x age + 11.41 x sex)\end{array}}$$ Probability (predicted Usual activities = 1) = 1–Probability (predicted Usual activities = 2)–Probability (predicted Usual activities = 3) predicted Usual activities, choosing if maximum predicted probability of Usual activities Probability (predicted Pain / discomfort = 2) = $$\frac{\begin{array}{c}exp(-3.29 + 0.03 x Q1 + 0.39 x Q3 - 0.02 x Q4 + 0.96 x Q6 + 0.72 x Q8 - 0.01 x age - 1.15 x sex)\end{array}}{\begin{array}{c}1 + \mathrm{exp}\left(-3.29 + 0.03\mathrm{ x Q}1 + 0.39\mathrm{ x Q}3 - 0.02\mathrm{ x Q}4 + 0.96\mathrm{ x Q}6 + 0.72\mathrm{ x Q}8 - 0.01\mathrm{ x age }- 1.15\mathrm{ x sex}\right) +\\ exp(-512.27 + 37.26 x Q1 - 6.14 x Q3 + 104.76 x Q4 + 12.01 x Q6 + 19.15 x Q8 - 2.05 x age - 30.83 x sex)\end{array}}$$ Probability (predicted Pain / discomfort = 3) = $$\frac{\begin{array}{c}exp(-512.27 + 37.26 x Q1 - 6.14 x Q3 + 104.76 x Q4 + 12.01 x Q6 + 19.15 x Q8 - 2.05 x age - 30.83 x sex)\end{array}}{\begin{array}{c}1 + \mathrm{exp}\left(-3.29 + 0.03\mathrm{ x Q}1 + 0.39\mathrm{ x Q}3 - 0.02\mathrm{ x Q}4 + 0.96\mathrm{ x Q}6 + 0.72\mathrm{ x Q}8 - 0.01\mathrm{ x age }- 1.15\mathrm{ x sex}\right) +\\ exp(-512.27 + 37.26 x Q1 - 6.14 x Q3 + 104.76 x Q4 + 12.01 x Q6 + 19.15 x Q8 - 2.05 x age -30.83 x sex)\end{array}}$$ Probability (predicted Pain / discomfort = 1) = 1–Probability (predicted Pain / discomfort = 2)–Probability (predicted Pain / discomfort = 3) predicted Pain / discomfort, choosing if maximum predicted probability of Pain / discomfort Probability (predicted Anxiety / depression = 2) = $$\frac{\begin{array}{c}exp(-2.78 - 0.34 x Q2 + 0.59 x Q3 + 0.31 x Q5 + 0.64 x Q6 + 0.59 x Q7 - 0.03 x age)\end{array}}{\begin{array}{c}1 + \mathrm{exp}\left(-2.78 - 0.34\mathrm{ x Q}2 + 0.59\mathrm{ x Q}3 + 0.31\mathrm{ x Q}5 + 0.64\mathrm{ x Q}6 + 0.59\mathrm{ x Q}7 - 0.03\mathrm{ x age}\right) +\\ exp(-216.48 - 4.28 x Q2 + 17.46 x Q3 + 7.08 x Q5 + 27.76 x Q6 + 28.40 x Q7 - 0.86 x age)\end{array}}$$ Probability (predicted Anxiety / depression = 3) = $$\frac{\begin{array}{c}exp(-216.48 - 4.28 x Q2 + 17.46 x Q3 + 7.08 x Q5 + 27.76 x Q6 + 28.40 x Q7 - 0.86 x age)\end{array}}{\begin{array}{c}1 + \mathrm{exp}\left(-2.78 - 0.34\mathrm{ x Q}2 + 0.59\mathrm{ x Q}3 + 0.31\mathrm{ x Q}5 + 0.64\mathrm{ x Q}6 + 0.59\mathrm{ x Q}7 - 0.03\mathrm{ x age}\right) +\\ exp(-216.48 - 4.28 x Q2 + 17.46 x Q3 + 7.08 x Q5 + 27.76 x Q6 + 28.40 x Q7 - 0.86 x age)\end{array}}$$ Probability (predicted Anxiety / depression = 1) = 1–Probability (predicted Anxiety / depression = 2)–Probability (predicted Anxiety / depression = 3) predicted Anxiety / depression, choosing if maximum predicted probability of Anxiety/ depression **Predicted utility** = 1–0.185–0.123 × predicted Mobility at level 2–0.272 × predicted Mobility at level 3–0.167 × predicted Self-care at level 2–0.276 × predicted Self-care at level 3–0.085 × predicted Usual activities at level 2–0.208 × predicted Usual activities at level 3–0.121 × predicted Pain / discomfort at level 2–0.261 × predicted Pain / discomfort at level 3–0.154 × predicted Anxiety / depression at level 2–0.282 × predicted Anxiety / depression at level 3–0.190 × Any dimension on level 3
Hoyle et al. M3 -OLS	**Predicted utility** = 0.9831816–0.0220703 × Q8–0.0418119 × Q6–0.0312604 × Q5–0.0260971 × Q3
Hoyle et al. M6 -OLS	Probability (predicted utility = 1) = $$\frac{\begin{array}{c}exp(-7.1242 + 0.2536439 x Q3 + 0.1635303 x Q4 + 0.4407377 x Q5 + \\ 0.3709118 x Q6 + 0.088462 x Q7 + 0.186294 x Q8)\end{array}}{\begin{array}{c}1 + exp(-7.1242 + 0.2536439 x Q3 + 0.1635303 x Q4 + 0.4407377 x Q5 +\\ 0.3709118 x Q6 + 0.088462 x Q7 + 0.186294 x Q8)\end{array}}$$ Probability (predicted utility > 0.5 < 1) = $$\frac{\begin{array}{c}exp(-1.4836 + 0.2536439 x Q3 + 0.1635303 x Q4 + 0.4407377 x Q5 +\\ 0.3709118 x Q6 + 0.088462 x Q7 + 0.186294 x Q8)\end{array}}{\begin{array}{c}1 + exp(-1.4836 + 0.2536439 x Q3 + 0.1635303 x Q4 + 0.4407377 x Q5 +\\ 0.3709118 x Q6 + 0.088462 x Q7 + 0.186294 x Q8)\end{array}}$$ Probability (predicted utility < 0.5) = 1- Probability (predicted utility = 1)- Probability (predicted utility > 0.5 < 1) **Predicted utility** = 1, if Maximum probability = Probability (predicted utility = 1) = 0.8150928–0.0114207 × Q3–0.0102185 × Q5–0.0270919 × Q6–0.0053779 × Q8, if Maximum probability = Probability (predicted utility > 0.5 < 1) = 0.3183917–0.00752 × CAT total scores, if Maximum probability = Probability (predicted utility < 0.5)
modified Hoyle et al. M3 -OLS	**Predicted utility** = 1.03670–0.01273 × Q8–0.06367 × Q6–0.03794 × Q5–0.01211 × Q3
modified Hoyle et al. M6 -OLS	Probability (predicted utility = 1) = $$\frac{\begin{array}{c}exp(-10.9642 + 0.2622 x Q3 + 0.3884 x Q4 + 0.8401 x Q5 + 1.0693 x Q6 + 0.1832 x Q7 + 0.1565 x Q8)\end{array}}{\begin{array}{c}1 + exp(-10.9642 + 0.2622 x Q3 + 0.3884 x Q4 + 0.8401 x Q5 + 1.0693 x Q6 + \\ 0.1832 x Q7 + 0.1565 x Q8)\end{array}}$$ Probability (predicted utility > 0.5 < 1) = $$\frac{\begin{array}{c}exp(-4.6737 + 0.2622 x Q3 + 0.3884 x Q4 + 0.8401 x Q5 + 1.0693 x Q6 + 0.1832 x Q7 + 0.1565 x Q8)\end{array}}{\begin{array}{c}\begin{array}{c}1 + exp(-10.9642 + 0.2622 x Q3 + 0.3884 x Q4 + 0.8401 x Q5 + 1.0693 x Q6 + \\ 0.1832 x Q7 + 0.1565 x Q8)\end{array}\end{array}}$$ Probability (predicted utility < 0.5) = 1–Probability (predicted utility = 1)–Probability (predicted utility > 0.5 < 1) **Predicted utility** = 1, if Maximum probability = Probability (predicted utility = 1) = 0.92209–0.01266 × Q3–0.01911 × Q5–0.03696 × Q6–0.02121 × Q8, if Maximum probability = Probability (predicted utility > 0.5 < 1) ** = **0.53984–0.01008 × CAT total scores, if Maximum probability = Probability (predicted utility < 0.5)
Lim et al. using CAT total scores	**Predicted utility** = 1.1376–0.0103 × CAT total scores–0.0020 × age
Lim et al. using CAT items	**Predicted utility** = 1.0661–0.0103 × Q3–0.0120 × Q4–0.0168 × Q5–0.0255 × Q6–0.0125 × Q8
modified Lim et al. using CAT total scores	**Predicted utility** = 1.26848–0.02159 × CAT total scores–0.00188 × age
modified Lim et al. using CAT items	**Predicted utility** = 1.05245–0.01137 × Q3–0.00810 × Q4–0.03702 × Q5–0.05993 × Q6–0.01286 × Q8

CAT questionnaires items: Q1 ~ 8 (Q1 = cough, Q2 = phlegm, Q3 = chest tightness, Q4 = breathlessness, Q5 = activities, Q6 = confidence, Q7 = sleep, Q8 = energy item.

**Table 2:** Comparison of predicted utility scores using the various models.

Total CAT score model-5 multinomial logistic regression and transform to utility						
Model	N	mean	Min	Max	MAE	RMSE
**Validation with full data set**						
	1176	0.944	0.100	1.000	0.0560	0.1222
**Validation by subgroup (severe stages by PFT)**						
stage 1	276	0.958	0.280	1.000	0.0435*	0.0971*
stage 2	635	0.958	0.280	1.000	0.0569	0.1276
stage 3	265	0.897	0.100	1.000	0.0668*	0.1322
**Validation by subgroup (utility)**						
utility: 0- < 0.25	6	0.372	0.100	0.860	0.2345*	0.3260*
utility: 0.25- < 0.5	28	0.737	0.194	1.000	0.3956	0.4240
utility: 0.5- < 0.75	156	0.788	0.100	1.000	0.1698*	0.2149*
utility: ≥ 0.75	986	0.979	0.280	1.000	0.0272	0.0690
**Validation by subgroup (CAT total scores)**						
0 ≤ CAT ≤ 10	730	1.000	1.000	1.000	0.0172	0.0655
11 ≤ CAT ≤ 20	384	0.905	0.466	1.000	0.1157	0.1770
21 ≤ CAT ≤ 30	60	0.548	0.194	0.676	0.1392*	0.1909*
31 ≤ CAT ≤ 40	2	0.100	0.100	0.100	0.2317	0.3277
Selected CAT items model-5 multinomial logistic regression and transform to utility						
**Validation with full data set**						
	1176	0.943	0.173	1.000	0.0538*	0.1181*
**Validation by subgroup (severe stages by PFT)**						
stage 1	276	0.975	0.342	1.000	0.0510	0.1093
stage 2	635	0.951	0.194	1.000	0.0489*	0.1175*
stage 3	265	0.891	0.173	1.000	0.0683	0.1280*
**Validation by subgroup (utility)**						
utility: 0- < 0.25	6	0.490	0.173	0.748	0.3519	0.4103
utility: 0.25- < 0.5	28	0.648	0.183	1.000	0.3073*	0.3499*
utility: 0.5- < 0.75	156	0.770	0.173	1.000	0.1731	0.2188
utility: ≥ 0.75	986	0.982	0.342	1.000	0.0259*	0.0675*
**Validation by subgroup (CAT total scores)**						
0 ≤ CAT ≤ 10	730	0.997	0.748	1.000	0.0167*	0.0600*
11 ≤ CAT ≤ 20	384	0.906	0.342	1.000	0.1025*	0.1640*
21 ≤ CAT ≤ 30	60	0.548	0.173	0.943	0.1889	0.2364
31 ≤ CAT ≤ 40	2	0.215	0.173	0.256	0.1904*	0.2236*

*Better perfomance for comparison of total CAT and selected CAT items models using the MAE and RMSE.

**Figure 1:**
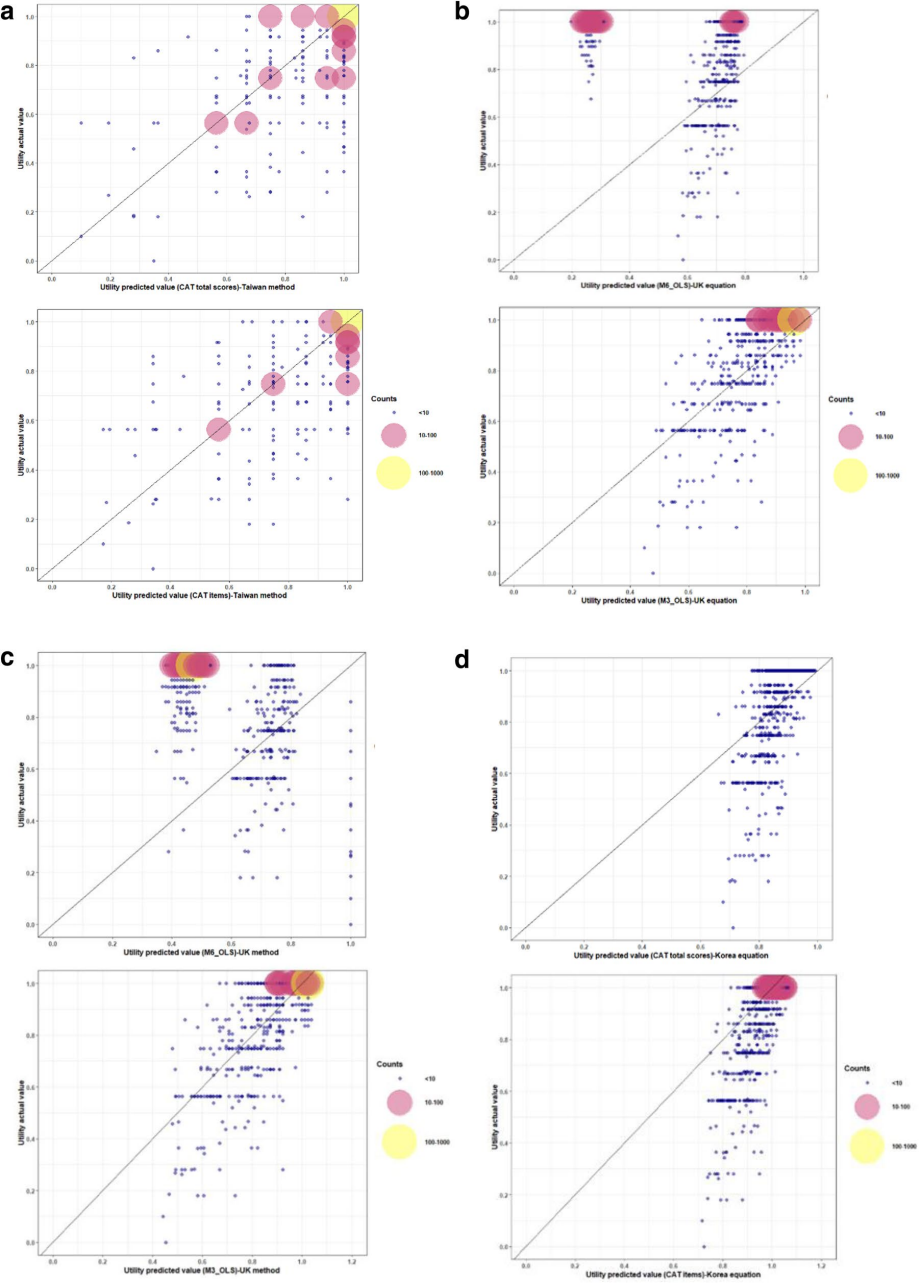

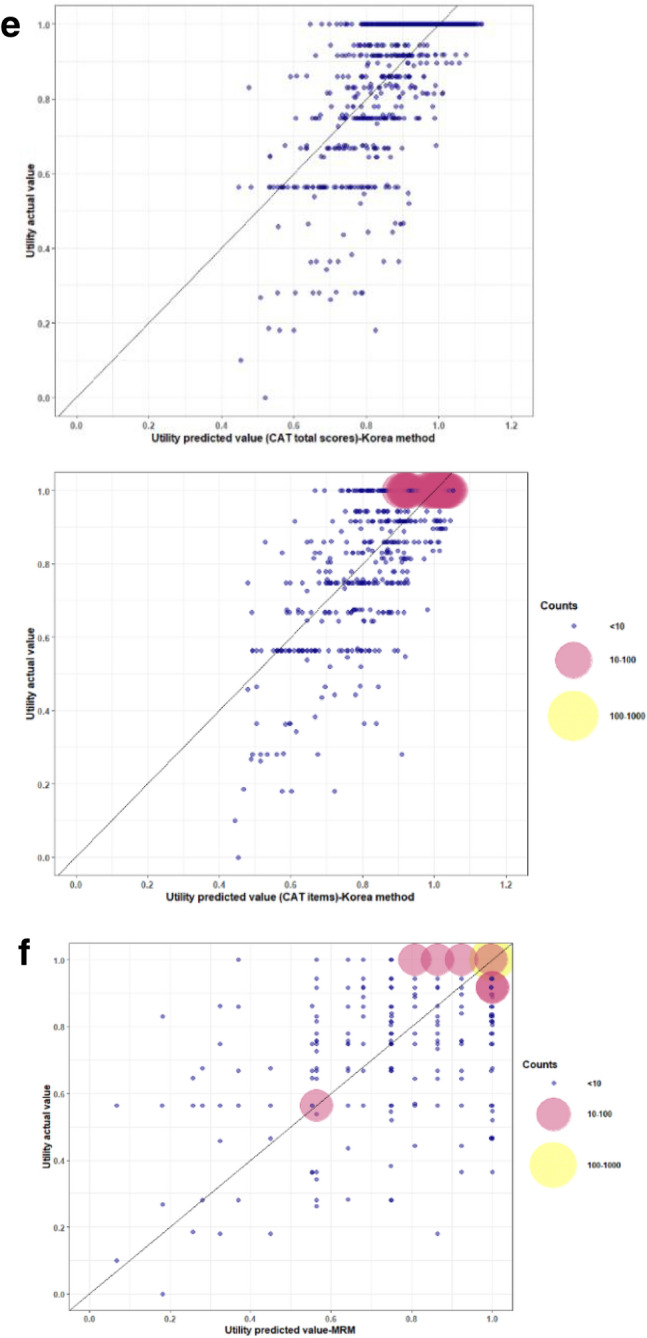
Bubble chart for actual and predicted utility. The real-predictive bubble chart presenting the distribution of the actual EQ-5D-3L utility with its predictive value based on the developed models. These charts revealed predicted utilities on the X axis and observed utilities on the Y axis. The bubble sizes and colors depict the predicted number of actual samples, where bigger bubbles mean a larger sample size. The colors of small, medium and large size bubbles were blue, pink and yellow color, respectively. When more bubbles are located adjacent to or on the diagonal line, this indicates a higher EQ-5D value prediction. Afterwards, acceptable fit requires that a greater number of large bubbles are located within a suitable margin along the diagonal line. (**a**) The real-predictive bubble chart using the formula based on CAT total scores and CAT items developed in this study. (**b**) The real-predictive bubble chart using the formula recommended by Hoyle et al. was applied to Taiwan datasets (M6_OLS and M3_OLS). (**c**) The real-predictive bubble chart presenting the predictive EQ-5D utility with the Model of modified Hoyle et.al., the model was developed by using the CAT scores in this study and equations based on OLS (ordinary least square) method from Hoyle et.al (M6_OLS and M3_OLS). (**d**) The real-predictive bubble chart presenting the predictive EQ-5D utility with the formula using the total CAT scores and CAT items recommended by Lim et al. for Taiwan datasets. (**e**) The real-predictive bubble chart presenting the predictive EQ-5D utility with the Model of modified Lim et.al., the model was developed by using the total CAT scores and CAT items in this study and equations based on OLS (ordinary least square) method from Lim et.al. (**f**) The real-predictive bubble chart presenting the predictive EQ-5D utility with the mean rank method, MRM recommended by Wee et al. for Taiwan datasets.

Comparing with the models developed by Hoyle et al., Lim et al. and Wee et al., non-inferior predictive effectiveness over the total and selected CAT items was found based on the MAE and RMSE results (Table 3). According to the real-predictive bubble charts, the method developed for the purposes of this study revealed comparable predictive capability to the other models.. Based on these bubble charts, all of the models had good predictive accuracy for the COPD patients with a better health status (Fig. 1). Figure 1a showed the real-predictive bubble chart using the formula developed in this study. The selected CAT items model led to obtaining a more precise prediction than the total CAT score model. The accuracy of the predictive model was better in the case of patients with higher EQ-5D utility. Figure 1b showed the real-predictive bubble chart using the formula recommended by Hoyle et al. applied to Taiwan datasets, and Fig. 1c showed the predictive EQ-5D utility with the model of modified Hoyle et.al., the model was developed by using the CAT score in this study and equations based on OLS (ordinary least square) method from Hoyle et.al. The model with the M6_OLS equation showed overestimation for lower utility (utility ≤ 0.5) patients and underestimation for near health (utility > 0.9) patients. The model with the M3_OLS equation had better predictive power than the M6_OLS equation for Taiwan datasets, but overestimated lower EQ-5D (utility ≤ 0.5) patients. Figure 1d showed the real-predictive bubble chart presenting the predictive EQ-5D utility with the formula recommended by Lim et al. for Taiwan datasets, and Fig. 1e showed the model of modified Lim et.al.. Both models showed that better predictive effectiveness was reported with the CAT items equation for patients with higher EQ-5D utilities. However, poor predictive power with overestimation was found for patients with lower EQ-5D utilities (utility ≤ 0.6) in the models with the CAT total scores and CAT items equations. Figure 1f showed the real-predictive bubble chart presenting the predictive EQ-5D utility with the mean rank method, recommended by Wee et al. for Taiwan datasets. The accuracy of the predictive model was similar to the developed model in this study (Fig. 1a). The overestimation for low utility and underestimation for near health patients in models developed by Hoyle et al. and Lim et al. was improved in the presented model and model developed by Wee et al.

**Table 3:** Comparison of predicted utility scores using the current developed models.

Developed models	N	mean	Min	Max	MAE	RMSE
**Present study**						
CAT total score model	1176	0.944	0.100	1.000	0.0560	0.1222
CAT selected items model	1176	0.943	0.173	1.000	0.0538	0.1181
**Hoyle et al**						
M3_OLS	1176	0.87	0.45	0.98	0.0918	0.1175
M6_OLS	1176	0.49	0.20	0.79	0.4590	0.5387
**Modified Hoyle et al**						
M3_OLS*	1176	0.92	0.44	1.04	0.0701	0.1032
M6_OLS*	1176	0.53	0.35	1.00	0.4354	0.4697
**Lim et al**						
CAT total scores	1176	0.89	0.66	0.99	0.1012	0.1312
CAT items	1176	0.97	0.72	1.07	0.0768	0.1323
**Modified Lim et al**						
CAT total scores*	1176	0.91	0.45	1.12	0.0818	0.1164
CAT items*	1176	0.92	0.44	1.05	0.0696	0.1027
Wee et al Mean Rank Method	1176	0.91	0.07	1.00	0.0643	0.1266

* Model of modified Hoyle et al. and Lim et al. were developed by using the CAT score in this study and equations based on OLS (ordinary least square) method from Hoyle et al. and Lim et al.

## Discussion

In this study, backward elimination with response mapping was carried out to develop the formula by which to transform the CAT scores into an EQ-5D score. Subsequently, estimating the utility weights of the EQ-5D was implemented with time trade-off method based on the EQ-5D scores. The performance of selected CAT items model was better in terms of estimation effectiveness than CAT total scores model.

The RMSE and MAE are standard measurements for choosing the best model among several models. Therefore, both the RMSE and MAE were used to evaluate their predicting ability for developed models in this study. Compared with the models in Hoyle et al.^9^ and Lim et al.^10^, comparable predictive effectiveness over selected CAT items was found based on the RMSE and MAE results.

However, previous models severely overestimate the EQ-5D utilities when the true utilities are relatively low^9,10^. By contrast, the model proposed in this study was more appropriate for the patient with COPD in Taiwan. From the bubble charts, overestimated EQ-5D utilities for the actual low utility value (< 0.5) were more distinct using the previous models within the datasets.

In the case of the model recommended by Holey et al., overestimated EQ-5D utility was reported for the COPD patients with poor health (utility < 0.7) or the extremely severe cases (CAT: 31 ~ 40). Also, underestimation of EQ-5D utility was noted in COPD patients with near health status (utility > 0.9). In the model proposed by Lim et al., there was also overestimation in predicting EQ-5D utility reported in patients at the very severe stage of COPD, and underestimation of the EQ-5D utility was reported in COPD patients at the mild stage.

In literature, OLS based approaches are popular for mapping disease-related measurements onto the EQ-5D utility^9,10^. The OLS algorithm using the CAT profile, the M3_OLS, was recommended by Hoyle et al. In a comparison with the MLR algorithm in this study, a higher RMSE was reported for the MLR model after adjusting the coefficient of the OLS algorithm using the data from this study. However, a lower MAE was noted when using the MLR model for the datasets in the present study. Another mapping algorithm for EQ-5D-3L utility prediction of COPD patients, the OLS1 and OLS3 models recommended by Lim et al., were also applied to the datasets in the present study. An even higher RMSE, but a lower MAE, were reported using the MLR model even after adjusting the coefficient of the OLS algorithm with the data from this study.. When ranking the mapping algorithms based on the MAEs and RMSEs, the model developed for this study was found to be comparable with developed models by Holey et al. and Lim et al., However, when comparing our model with models from Holey et al. and Lim et al. by the real-predictive bubble charts, our model had better predictive effectiveness among patients with poor utility and near health in this study t.

This model recommended in the present study exhibited better predictive power than other models in terms of mapping EQ-5Q utility from the CAT for COPD patients in Taiwan. However, overestimated EQ-5D utilities were still observed for patients with poorer health status (actual utility < 0.5), because of small sample size. Therefore, the large prediction bias may have been due to the small sample size of very severe COPD patients or due to the heterogeneity arising because of COPD severity. This phenomenon calls attention to the fact that choosing the best model by merely considering one or two indices may result in an unexpected result.

The time trade-off values of coefficients in estimating quality weight of EQ-5D health states differ from country to country^20,21^. This difference might be due to the sociodemographic background of the respondents and methodological differences in studies^21^. And then, the difference could contribute not only one appropriate model for developing mapping algorithms. For example, the respondents in South Korea put more weight on mobility and self-care domains than the other three dimensions^22^. For UK respondents, the pain/discomfort domain was considered to be more important than the other four dimensions^23^. As for Taiwanese, the respondents devote their mind to the anxiety/depression domain more than others^16^.

Apart from the MAE and RMSE results, according the real-predictive bubble charts, the method developed from this study reveals comparable predictive capability to the models developed by Hoyle et a. in UK and Lim et al. in South Korea. The models from the UK, South Korea, and Taiwan group all presented well accuracy of prediction over the COPD patients with better health status. By contrast, poorer predictive performance was revealed under the models of UK and South Korea in the COPD patients with poorer utility and near health than the present models. We have tried to apply MRM, developed by Wee et al. as the other method for developing a predictive model of mapping EQ-5D utilities from CAT in this study. The predictive ability of the MRM model was better than the models of UK and South Korea and was similar to the present models base on real-predictive bubble charts. Therefore, other methods even developed not for mapping EQ-5D utilities from CAT in original, should be tried in the future to get the best predictive model for different populations.

## Conclusions

Response mapping with MLR model and model using MRM method has comparable performance with OLS model for predicting EQ-5D utility from CAT in Taiwan. In addition, the overestimation for low utility patients and underestimation for near health in previous developed OLS models was improved in the presented models and model using MRM method. However, it is better to administer both CAT and EQ-5D-3L if the cost-utility analysis is planned for clinical trial or study; the mapping should be the last resort as it can only give an approximate utility value.

## Supplementary Information

Supplementary Information.

## Data Availability

Full data set are not available publicly currently for protecting patient privacy. However, the data can be obtained through a reasonable request to the corresponding author.
