# Supplementary material for: Mapping algorithms for predicting EuroQol-5D-3L utilities from the assessment test of chronic obstructive pulmonary disease
Source: Sci Rep. 2022 Dec 3;12:20930. doi: 10.1038/s41598-022-24956-2 (PMC9719462; doi:10.1038/s41598-022-24956-2)
Supplement: Supplementary file 1 — Supplementary Information. [file 41598_2022_24956_MOESM1_ESM.docx]

**Supplemental material for**

**Mapping Algorithms for Predicting EuroQol-5D-3L Utilities from the Assessment Test of chronic obstructive pulmonary disease**

Chun-Hsiang Yu^1^, Sheng-Mao Chang^2^, Chih-Hui Hsu^3^, Sheng-Han Tsai^4^, Xin-Min Liao^1^, Chang-Wei Chen^1^, Ching-Hsiung Lin^5^, Jung-Der Wang^6^, Tzuen-Ren Hsiue^1^, and Chiung-Zuei Chen^1^*

Authors’ affiliation

1. Division of Pulmonary Medicine, Department of Internal Medicine, National Cheng Kung University Hospital, College of Medicine, National Cheng Kung University, Tainan, Taiwan

2. Department of Statistics, National Taipei University

3. Clinical Medicine Research Center, National Cheng Kung University Hospital, Tainan, Taiwan

4. Division of General Medicine, Department of Internal Medicine, National Cheng Kung University Hospital, College of Medicine, National Cheng Kung University, Tainan, Taiwan

5. Division of Chest Medicine, Department of Internal Medicine, Changhua Christian Hospital, Changhua, Taiwan

6. Department of Public Health, College of Medicine, National Cheng Kung University, Tainan, Taiwan

Corresponding author full contact details:

Name: Chiung-Zuei Chen

Address: No. 138 Sheng-Li Road

Post code: 704

City: Tainan

Country: Taiwan

Email: [chen96@mail.ncku.edu.tw](mailto:chen96@mail.ncku.edu.tw)

**Supplementary Tables**

supplementary Table 1.

**Comparison of demographic and clinical characteristics between patients randomly allocated into the two groups**

|  | **Training group**  **(N=160)** | **Validation group**  **(N=163)** | **p-value** ^*^ |
| --- | --- | --- | --- |
| **Variables** | **n (%) / mean ± SD** | **n (%) / mean ± SD** |  |
| **Gender** |  |  | 0.537 |
| Male | 151 (94.38) | 150 (92.02) |  |
| Female | 9 (5.63) | 13 (7.98) |  |
| **Age** | 72.67 ± 10.34 | 72.43 ± 9.99 | 0.831 |
| **Age group** |  |  | 0.786 |
| <50 | 2 (1.25) | 1 (0.61) |  |
| 50-64 | 36 (22.50) | 37 (22.70) |  |
| 65-79 | 78 (48.75) | 86 (52.76) |  |
| ≥80 | 44 (27.50) | 39 (23.93) |  |
| **BMI** | 24.32 ± 5.02 | 24.01 ± 4.63 | 0.569 |
| **BMI group** |  |  | 0.812 |
| <18 | 8 (5.00) | 11 (6.75) |  |
| 18-<24 | 72 (45.00) | 78 (47.85) |  |
| 24-<27 | 38 (23.75) | 34 (20.86) |  |
| ≥27 | 42 (26.25) | 40 (24.54) |  |
| **mMRC** |  |  | 0.921 |
| 0 | 4 (2.50) | 2 (1.23) |  |
| 1 | 38 (23.75) | 39 (23.93) |  |
| 2 | 73 (45.63) | 72 (44.17) |  |
| 3 | 35 (21.88) | 39 (23.93) |  |
| 4 | 10 (6.25) | 11 (6.75) |  |
| **Smoking** |  |  | 0.967 |
| Never smoking | 21 (13.13) | 23 (14.11) |  |
| Quit smoking | 105 (65.63) | 106 (65.03) |  |
| Still smoking | 34 (21.25) | 34 (20.86) |  |
| **FEV1 (% predicted)** | 52.37 ± 11.88 | 50.88 ± 12.12 | 0.265 |
| **Pulmonary stage** |  |  | 0.571 |
| ≥ 80 (mild) | 44 (27.50) | 35 (21.47) |  |
| 50-80 (moderate) | 76 (47.50) | 87 (53.37) |  |
| 30-50 (severe) | 33 (20.63) | 32 (19.63) |  |
| <30 (very severe) | 7 (4.38) | 9 (5.52) |  |

^*^ The chi-square homogeneity test was used to test the distribution of categorical variables (Gender, Age group, BMI group, mMRC, Smoking, Pulmonary stage) between groups. The student’s t-test was used to test the distribution of continuous variables (Age, BMI, FEV1 (% predicted)) between groups.

supplementary Table 2

**Generalized estimating equations (GEE) for CAT mapping to EQ5D (Multinomial logistic regression) for model development**

- 1. **Mobility (total CAT score)**

| Variables | MOB=2 vs MOB=1 | |  | MOB=3 vs MOB=1 | |
| --- | --- | --- | --- | --- | --- |
|  | Estimate ± SE | p-value |  | Estimate ± SE | p-value |
| Intercept | -13.79±1.58 | <0.001 |  | -25.27±3.54 | <0.001 |
| CAT total scores | 0.37±0.03 | <0.001 |  | 0.48±0.06 | <0.001 |
| Age | 0.10±0.02 | <0.001 |  | 0.18±0.04 | <0.001 |
| Male vs. Female | 0.64±0.36 | 0.074 |  | -1.52±1.26 | 0.228 |

**1-2. Mobility (selected CAT items)**

| Variables | MOB=2 vs MOB=1 | |  | MOB=3 vs MOB=1 | |
| --- | --- | --- | --- | --- | --- |
|  | Estimate ± SE | p-value |  | Estimate ± SE | p-value |
| Intercept | -10.90±1.20 | <0.001 |  | -22.18±3.68 | <0.001 |
| CAT items |  |  |  |  |  |
| Phlegm | -0.14±0.11 | 0.200 |  | -0.01±0.45 | 0.980 |
| Chest tightness | -0.14±0.10 | 0.175 |  | -0.17±0.41 | 0.680 |
| Breathlessness | 0.59±0.10 | <0.001 |  | 0.51±0.35 | 0.139 |
| Activity at home | 0.76±0.12 | <0.001 |  | 1.41±0.39 | <0.001 |
| Confidence | 0.84±0.12 | <0.001 |  | 1.42±0.35 | <0.001 |
| Energy | 0.39±0.18 | 0.028 |  | -0.70±0.48 | 0.143 |
| Age | 0.07±0.01 | <0.001 |  | 0.15±0.04 | 0.001 |

**1-3.** **Self-care (total CAT score)**

| Variables | SELFC=2 vs SELFC=1 | |  | SELFC=3 vs SELFC=1 | |
| --- | --- | --- | --- | --- | --- |
|  | Estimate ± SE | p-value |  | Estimate ± SE | p-value |
| Intercept | -10.41±1.01 | <0.001 |  | -18.28±1.75 | <0.001 |
| CAT total scores | 0.38±0.02 | <0.001 |  | 0.49±0.05 | <0.001 |
| Age | 0.06±0.01 | <0.001 |  | 0.11±0.02 | <0.001 |
| Male vs. Female | 0.95±0.40 | 0.018 |  | 0.24±1.28 | 0.851 |

**1-4. Self-care (selected CAT items)**

| Variables | SELFC=2 vs SELFC=1 | |  | SELFC=3 vs SELFC=1 | |
| --- | --- | --- | --- | --- | --- |
|  | Estimate ± SE | p-value |  | Estimate ± SE | p-value |
| Intercept | -8.62±1.13 | <0.001 |  | -16.45±2.54 | <0.001 |
| CAT items |  |  |  |  |  |
| Phlegm | -0.04±0.13 | 0.771 |  | -0.08±0.31 | 0.796 |
| Activity at home | 1.44±0.14 | <0.001 |  | 2.16±0.26 | <0.001 |
| Confidence | 0.80±0.13 | <0.001 |  | 0.98±0.25 | <0.001 |
| Sleep | 0.06±0.14 | 0.699 |  | -0.05±0.28 | 0.848 |
| Age | 0.03±0.01 | 0.019 |  | 0.08±0.02 | <0.001 |
| Male vs. Female | 1.39±0.59 | 0.019 |  | 1.22±1.58 | 0.440 |

**1-5.** **Usual activities (total CAT score)**

| Variables | UACT=2 vs UACT =1 | |  | UACT=3 vs UACT=1 | |
| --- | --- | --- | --- | --- | --- |
|  | Estimate ± SE | p-value |  | Estimate ± SE | p-value |
| Intercept | -11.67±1.00 | <0.001 |  | -20.99±1.95 | <0.001 |
| CAT total scores | 0.45±0.03 | <0.001 |  | 0.63±0.05 | <0.001 |
| Age | 0.07±0.01 | <0.001 |  | 0.12±0.02 | <0.001 |
| Male vs. Female | 0.46±0.44 | 0.296 |  | 0.73±1.25 | 0.559 |

**1-6. Usual activities (selected CAT items)**

| Variables | UACT=2 vs UACT =1 | |  | UACT=3 vs UACT=1 | |
| --- | --- | --- | --- | --- | --- |
|  | Estimate ± SE | p-value |  | Estimate ± SE | p-value |
| Intercept | -9.43±1.00 | <0.001 |  | -17.88±2.10 | <0.001 |
| CAT items |  |  |  |  |  |
| Chest tightness | 0.27±0.12 | 0.019 |  | 0.29±0.18 | 0.114 |
| Breathlessness | 0.49±0.11 | <0.001 |  | 0.36±0.25 | 0.156 |
| Activity at home | 1.21±0.13 | <0.001 |  | 2.27±0.32 | <0.001 |
| Confidence | 0.97±0.13 | <0.001 |  | 1.21±0.34 | <0.001 |
| Sleep | 0.23±0.14 | 0.109 |  | 0.14±0.23 | 0.532 |
| Age | 0.05±0.01 | <0.001 |  | 0.08±0.02 | <0.001 |
| Male vs. Female | 0.70±0.36 | 0.049 |  | 1.47±1.10 | 0.179 |

**1-7. Pain/ Discomfort (total CAT score)**

| Variables | PAIN=2 vs PAIN=1 | |  | PAIN=3 vs PAIN=1 | |
| --- | --- | --- | --- | --- | --- |
|  | Estimate ± SE | p-value |  | Estimate ± SE | p-value |
| Intercept | -6.49±0.78 | <0.001 |  | -19.91±5.46 | <0.001 |
| CAT total scores | 0.31±0.02 | <0.001 |  | 0.69±0.11 | <0.001 |
| Age | 0.01±0.01 | 0.327 |  | 0.02±0.06 | 0.704 |
| Male vs. Female | -0.66±0.41 | 0.111 |  | 9.87±0.88 | <0.001 |

**1-8. Pain/ Discomfort (selected CAT items)**

| Variables | PAIN=2 vs PAIN=1 | |  | PAIN=3 vs PAIN=1 | |
| --- | --- | --- | --- | --- | --- |
|  | Estimate ± SE | p-value |  | Estimate ± SE | p-value |
| Intercept | -4.40±0.89 | <0.001 |  | -27.97±5.12 | 0.001 |
| CAT items |  |  |  |  |  |
| Phlegm | 0.08±0.12 | 0.466 |  | 0.63±0.64 | 0.320 |
| Chest tightness | 0.46±0.10 | <0.001 |  | 0.70±0.67 | 0.297 |
| Breathlessness | 0.08±0.09 | 0.409 |  | 0.61±1.16 | 0.602 |
| Confidence | 0.84±0.09 | <0.001 |  | 1.89±0.42 | <0.001 |
| Energy | 0.73±0.14 | <0.001 |  | 1.73±0.64 | 0.007 |
| Age | 0.002±0.01 | 0.885 |  | -0.01±0.09 | 0.906 |
| Male vs. Female | -0.66±0.42 | 0.122 |  | 9.87±0.93 | <0.001 |

**1-9.** **Anxiety/ Depression (total CAT score)**

| Variables | MOOD=2 vs MOOD=1 | |  | MOOD=3 vs MOOD=1 | |
| --- | --- | --- | --- | --- | --- |
|  | Estimate ± SE | p-value |  | Estimate ± SE | p-value |
| Intercept | -4.22±0.87 | <0.001 |  | -22.02±3.57 | <0.001 |
| CAT total scores | 0.28±0.02 | <0.001 |  | 0.68±0.19 | 0.001 |
| Age | -0.01±0.01 | 0.211 |  | -0.09±0.04 | 0.016 |
| Male vs. Female | -0.52±0.37 | 0.157 |  | 9.81±1.13 | <0.001 |

**1-10. Anxiety/ Depression (selected CAT items)**

| Variables | MOOD=2 vs MOOD=1 | |  | MOOD=3 vs MOOD=1 | |
| --- | --- | --- | --- | --- | --- |
|  | Estimate ± SE | p-value |  | Estimate ± SE | p-value |
| Intercept | -3.30±0.91 | <0.001 |  | -11.31±5.00 | 0.024 |
| CAT items |  |  |  |  |  |
| Phlegm | -0.11±0.12 | 0.349 |  | -0.42±0.38 | 0.266 |
| Chest tightness | 0.48±0.11 | <0.001 |  | 0.85±0.41 | 0.039 |
| Activity at home | 0.39±0.12 | 0.001 |  | 1.96±0.57 | 0.001 |
| Confidence | 0.55±0.12 | <0.001 |  | 1.42±1.12 | 0.206 |
| Sleep | 0.54±0.11 | <0.001 |  | 2.05±0.46 | <0.001 |
| Age | -0.02±0.01 | 0.088 |  | -0.15±0.08 | 0.067 |

Multinomial logistic regression is used to evaluate the effect of those included variables over the different levels of mobility (MOB), self-care (SELFC), usual activities (UAUC), pain/discomfort (PAIN), and anxiety/depression (MOOD) in the datasets of development group. These factors include CAT total scores, age, and gender. Generalized estimating equation (GEE) is used for CAT mapping to EQ5D. The quasi-information criterion (QIC) is adjusted for regression.

**Supplementary Figures**


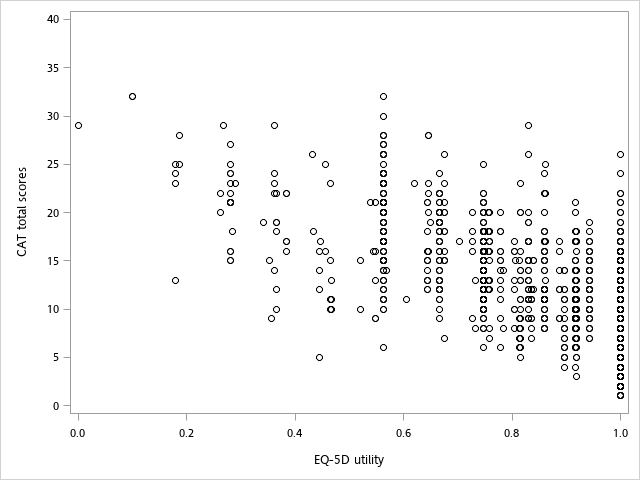
Supplementary Figure S1

Figure S1. Scatter graph of EQ-5D-3L utilities and CAT total scores. The darker circles represent higher data point density. A negative correlation (-0.69) between EQ-5D-3L utility and CAT total score was observed.

Supplementary Figure S2


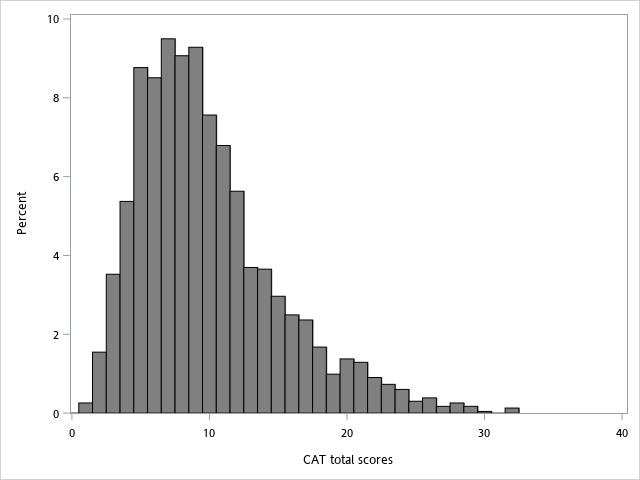

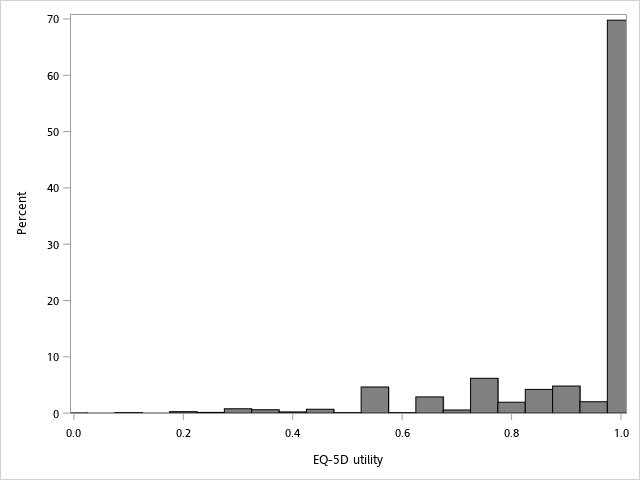


Figure S2. Distributions of EQ-5D-3L utilities and CAT total scores. The largest cluster was located at EQ-5D utility=1: n=1624 (69.79% of observations). The other clusters were 0 ≤ EQ-5D < 0.5: n=65 (2.79%) and 0.5 ≤ EQ-5D < 1: n=638 (27.42%).

CAT, COPD assessment test; COPD, chronic obstructive pulmonary disease; EQ-5D, EuroQol five-dimensional questionnaire.
